# A Magnetically Driven Amoeba‐Like Nanorobot for Whole‐Process Active Drug Transport

**DOI:** 10.1002/advs.202204793

**Published:** 2023-01-25

**Authors:** Yueqiang Zhu, Yonghong Song, Ziyang Cao, Liang Dong, Song Shen, Yang Lu, Xianzhu Yang

**Affiliations:** ^1^ Guangzhou First People's Hospital School of Biomedical Sciences and Engineering South China University of Technology Guangzhou International Campus Guangzhou 511442 P.R. China; ^2^ National Engineering Research Center for Tissue Restoration and Reconstruction and Guangdong Province Key Laboratory of Biomedical Engineering South China University of Technology Guangzhou Guangdong 510006 P. R. China; ^3^ Anhui Province Key Laboratory of Advanced Catalytic Materials and Reaction Engineering School of Chemistry and Chemical Engineering Intelligent Interconnected Systems Laboratory of Anhui Province Hefei University of Technology Hefei 230009 P.R. China; ^4^ Key Laboratory of Biomedical Materials and Engineering of the Ministry of Education South China University of Technology Guangzhou 510006 P. R. China

**Keywords:** active drug transport, deformability, magnetic field application, nanomedicines, tumor penetration

## Abstract

The passive diffusion performance of nanocarriers results in inefficient drug transport across multiple biological barriers and consequently cancer therapy failure. Here, a magnetically driven amoeba‐like nanorobot (amNR) is presented for whole‐process active drug transport. The amNR is actively extravasated from blood vessels and penetrated into deep tumor tissue through a magnetically driven deformation effect. Moreover, the acidic microenvironment of deep tumor tissue uncovers the masked targeting ligand of amNR to achieve active tumor cell uptake. Furthermore, the amNR rapidly releases the encapsulated doxorubicin (DOX) after alternating magnetic field application. The amNRs eventually deliver DOX into ≈92.3% of tumor cells and completely delay tumor growth with an inhibition rate of 96.1%. The deformable amNRs, with the assistance of magnetic field application, provide a facile strategy for whole‐process active drug transport.

## Graphical abstract

Magnetically‐driven amoeba‐like nanorobot for active drug delivery in the whole process is presented. Deformable amNR with a unique polyphosphate core is actively penetrated into deep tumor tissue under the assistance of a guiding magnetic field and achieved enhanced cell uptake and controlled drug release under the trigger of tumor acidic microenvironment and alternating magnetic field.

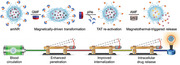

## 1 Introduction

Insufficient drug concentrations in tumor cells are considered one of the main causes of cancer therapy failure.^[^ ^1^, ^2^, ^3^ ^]^ Although significant progress has been made in nanomedicine‐mediated drug delivery, the transport efficiency is still unsatisfactory since almost all approved anticancer nanomedicines have failed to improve survival in clinical trials.^[^ ^4^, ^5^ ^]^ One critical reason is that all of these approved nanomedicines are unable to passively transport the drugs across a series of biological barriers throughout the processes of blood circulation, tumor accumulation, tumor penetration, cellular internalization, and intracellular release. For example, the passive transvascular transport of nanoparticles (NPs) relies on a large concentration gradient of NPs from the circulation toward the tumor tissue.^[^ ^6^ ^]^ However, NPs are quickly opsonized and cleared by the mononuclear phagocyte system, which ultimately results in inefficient tumor accumulation.^[^ ^7^ ^]^ Next, the elevated interstitial fluid pressure, high extracellular matrix density, and disorganized vascular networks severely prevent the passive diffusion of NPs into the deep tumor tissue, especially those with sizes up to 100 nm.^[^ ^8^, ^9^ ^]^ For example, Doxil (a liposomal doxorubicin (DOX) with a diameter of ≈100 nm, which has higher tumor accumulation than free DOX), is predominantly localized near the vasculature regions, resulting in low therapeutic efficacy.^[^ ^10^, ^11^ ^]^ Furthermore, the passive and slow intracellular release of the drugs from the NPs fails to achieve an effective drug concentration to kill tumor cells.^[^ ^12^, ^13^ ^]^

To overcome these hurdles for passive nanomedicines, active drug delivery systems have been utilized to increase drug transport efficacy. For example, various live cell‐based hitchhiking formulations have been developed to transport NPs due to their “self by host” natural properties and intrinsic targeting capabilities,^[^ ^14^, ^15^ ^]^ and anaerobe‐carrying NPs can selectively deliver them into hypoxic areas in the tumor core.^[^ ^16^, ^17^ ^]^ In addition, self‐propelled micro/nanomotors with autonomous movement ability allow these NPs to actively move to the desired areas for enhanced tumor accumulation and penetration.^[^ ^18^, ^19^, ^20^, ^21^ ^]^ However, these systems can only achieve active drug transport in a partial delivery process. According to the Cannikin law, overall drug transport efficiency is always limited by the shortest board, and an ideal nanomedicine should actively transport drugs throughout the entire delivery process.

**Figure 1:**
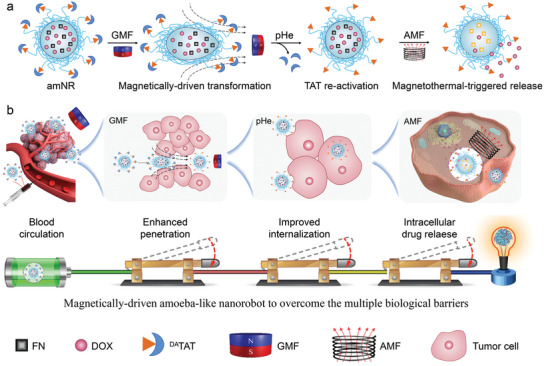
a) Schematic of the structural changes of amNR under the application of a magnetic field. b) Schematic illustration of the amoeba‐like nanorobot (amNR) for whole‐process active drug transport. First, the amNRs actively extravasated and penetrated into deep tumor tissue via magnetically driven deformation. Then, the acidic microenvironment of the deep tumor tissue uncovered the masked ligand (transactivator of transcription (TAT) peptide) of the amNRs for active tumor cell uptake. Next, the AMF triggered intracellular DOX release from the amNRs. Finally, the deformable amNRs achieved whole‐process active drug transport for superior antitumor efficiency with the assistance of magnetic field application.

Inspired by the distinctive deformation capability of an amoeba to actively bypass obstacles,^[^ ^22^ ^]^ we proposed to develop a magnetically driven amoeba‐like nanorobot (named amNR) for whole‐process active drug transport (**Figure** **1**). Under guiding magnetic field (GMF) actuation, the deformable amNR actively extravasated from blood vessels and deeply penetrated into the tumor. Moreover, the acidic environment in the deep tumor tissue uncovered the masked targeting ligand of amNR for active tumor cell uptake. Furthermore, the amNR rapidly released the encapsulated DOX, which entered the cell nucleus through alternating magnetic field (AMF) induction. Overall, the deformable amNRs achieved whole‐process active drug transport under magnetic actuation, enabling complete tumor growth inhibition after five treatments.

## 2 Results

### 2.1 Design and Characterization of Magnetically Driven amNR

**Figure 2:**
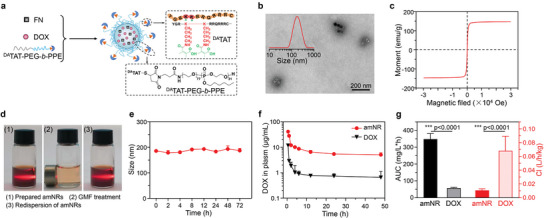
Preparation and characterization of amNRs. a) Schematic illustrating the components of the amNRs. b) Representative TEM images and size distribution (inset) of the amNRs, demonstrating a size of ≈185 nm. c) Magnetization curve of the amNRs. d) Orientational actuation and redispersion of prepared amNRs under GMF. The solutions were irradiated by a light beam to show the Tyndall scatter phenomenon. e) Changes in amNR size in culture medium containing 10% FBS. f) Plasma pharmacokinetic curves of amNR and DOX after intravenous injection (n = 3). g) The key pharmacokinetic parameters AUC and Cl of amNR and DOX. Data are shown as mean ± S.D. n = 3. ***p < 0.001.

**Figure** **2**a schematically illustrates the preparation of the drug‐loaded amNR by the tumor acidity‐sensitive transactivator of transcription (TAT) peptide‐decorated poly(ethylene glycol)‐*b*‐polyphosphoester (^DA^TAT‐PEG‐*b*‐PPE). The amNR core is made of a polyphosphoester (PPE), which endowed the amNR with deformability characteristics at room temperature. A ferrimagnetic nanocube (FN) and DOX were encapsulated into the amNR core, serving as the magnetically driven component and model drug, respectively. The amNR surface was decorated with the acid‐sensitive ligand peptide ^DA^TAT, whose targeting capability was masked in the circulation but uncovered in the acidic tumor microenvironment. The UV–vis spectrum of the amNRs in Figure S1 (Supporting Information) displayed characteristic peaks for FN and DOX, indicating successful amNR preparation. In addition, the DOX loading content for amNR at an optimal feed ratio of (2:20) used in subsequent experiments was 3.59 ± 0.34% (Figure S2, Supporting Information). And, the amNRs had an average hydrodynamic diameter of ≈185 nm and a sphere‐like morphology (Figure 2b). Hysteresis curve analysis showed that the amNRs exhibited a relatively high saturation magnetization of 147.2 emu g^−1^ due to the encapsulated FN (Figure 2c). Therefore, amNRs possessed outstanding magnetic response ability under GMF treatment (Figure 2d). Additionally, the amNRs exhibited good colloidal stability after incubation with fetal bovine serum (FBS) for 72 h (Figure 2e) and also possessed satisfactory size stability during the dilution process (Figure S3, Supporting Information).

The masked TAT peptide on the amNRs minimizes the nonspecific uptake by nontumor cells (e.g., macrophages in peripheral blood monocytes), which can prolong the circulation time.^[^ ^23^ ^]^ The pharmacokinetic curve (Figure 2f) of the amNRs demonstrated prolonged blood circulation in comparison to free DOX, which was further confirmed by calculating the area under the curve (AUC) and clearance (Cl) of the drug (Figure 2g).

### 2.2 GMF Drives the amNRs to Actively Penetrate into the Whole Tumor Tissue

**Figure 3:**
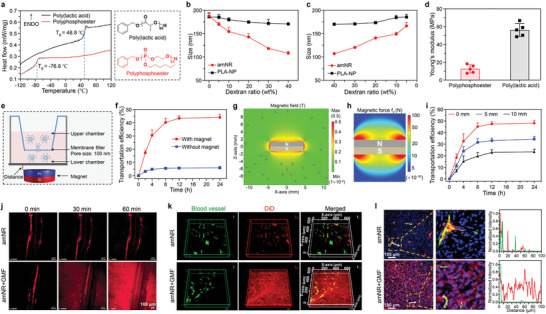
The distinctive deformability of the amNRs ensured their magnetic field‐driven deep penetration into tumor tissue. a) Differential scanning calorimetry (DSC) traces of polyphosphoester (PPE) and poly(lactic acid) (PLA). The glass transition temperatures (*T*_g_s) of PPE and PLA were −76.8 °C and 48.8 °C, respectively. The right panel shows the chemical structures of PPE and PLA. b,c) Hydrodynamic diameters of amNRs (PPE core) and PLA‐NPs (PLA core) in dextran solutions with different concentrations. d) The Young's modulus was determined by atomic force microscopy of PPE and PLA. e) Schematic illustration of the 100 nm filter membrane fixed Transwell system to evaluate the deformability of the amNRs under a GMF. f) Transportation efficiency of amNRs across a 100 nm membrane filter under a GMF. g) Representative finite element modeling of the magnetic field magnitude B plots for the 8 mm diameter and 3 mm thick NdFeB cylindrical magnet. h) Simulated distribution of the magnetic force on the amNRs in the z‐direction. The simulated magnetic force calculations were based on the method reported in the literature.^[^ ^26^ ^]^ i) Transportation efficiency of amNRs across a 100 nm membrane filter at different distances from the cylindrical magnet. The distance was considered the length from the surface of the cylindrical magnet to the bottom of the Transwell. j) Real‐time intravital observation of the distribution of DiD‐labeled amNRs (red) in the dorsal skin fold window chamber tumor model after intravenous administration. k) Spatial distribution of DiD‐labeled amNRs (red) in tumor tissue. l) The intratumoral distribution of DiD‐labeled amNRs under a GMF. Blood vessels: green; the cellular nuclei: blue. The fluorescence colocalization analysis of DiD‐labeled amNRs (red line) and blood vessels (green line) was shown in the right section.

To simulate the distinctive deformability of amoebae, soft PPE was utilized to construct the amNR core because the PPE had a much lower glass transition temperature (−76.8 °C) than the commonly used poly(lactic acid) (**Figure** **3**a). On this basis, we evaluated the deformability of amNRs and poly(lactic acid) core nanoparticles (PLA‐NPs) by measuring their sizes in different osmotic solutions. Figure 3b demonstrated that the size of the amNRs gradually decreased with increasing osmotic pressure after the addition of dextran. With a decrease in the osmotic pressure by adding water, the size of the amNRs gradually recovered (Figure 3c). In contrast, the change in PLA‐NP size was negligible. To reveal the underlying reason for these observations, Young's modulus of PPE was first determined by atomic force microscopy and was much lower than that of poly(lactic acid) (Figure 3d). The above results confirmed that the soft PPE endowed the amNRs with good deformability.

Next, to assess whether deformable amNRs can pass through the small interstitium under an applied extrusion force, polycarbonate films with different pore sizes were employed (Figure S4a, Supporting Information). As shown in Figure S4b–e (Supporting Information), amNRs (≈185 nm) can efficiently cross through 100, 80, and even 50 nm filters. More importantly, the size of filtered amNRs was clearly higher than the pore size of the filter. Meanwhile, amNR still maintained good size stability after passing through the filter (Figure S5, Supporting Information). In contrast, PLA‐NPs could not pass through the 100 nm pore filter (Figure S6, Supporting Information). Taken together, these results further confirmed that the soft PPE core endowed the amNRs with their distinctive deformability.

Furthermore, to validate whether a GMF could drive deformable amNR to cross the small interstitium, a 100 nm filter membrane was fixed at the chamber of a Transwell system, and then a NdFeB cylindrical magnet was placed under the system (Figure 3e). As shown in Figure 3f, ≈44.2% of the amNRs passed through the 100 nm filter after GMF application, while without the GMF, their passage was negligible (lower than 6%). More interestingly, the size of the amNRs decreased slightly after passing through the 100 nm filter (Figure S7, Supporting Information), which indicated that the magnetic force can drive the deformable amNR to pass through the small interstitium. To quantify the magnetic force experienced by the amNRs, representative finite element modeling of the magnetic field magnitude (B) was performed for the NdFeB cylindrical magnet used (Figure 3g). Subsequently, the simulated distribution of the magnetic force on the amNRs in the z direction was calculated. As shown in Figure 3h, the force exerted on the amNRs in the magnetic field sharply decreased with a gradual increase in the distance from the cylindrical magnet. Since effective magnetic actuation for the amNRs was dependent on the magnitude of magnetic force,^[^ ^24^, ^25^ ^]^ we experimentally analyzed the transportation efficiency‐distance relationship (Figure 3i). The results showed that the transportation efficiency of the amNRs through the 100 nm filter membrane decreased with increasing distance between the Transwell system and magnet (reduction in magnetic force on the amNRs). The above results showed that the amNRs could pass through small obstacles by deformation under the actuation of magnetic force.

Encouraged by their distinctive deformability, we further assessed whether the GMF could drive the amNRs to actively penetrate into deep tumor tissue. A dorsal skin fold window chamber model of the MDA‐MB‐231 tumor was constructed for real‐time intravital observation of the intratumoral distribution of amNRs (Figure S8, Supporting Information). One hour after the injection of DiD‐labeled amNRs, the mice were anesthetized and placed on the electric stage of a spinning‐disk confocal microscope for intratumoral visualization. Figure 3j and Movies S1,S2 (Supporting Information) demonstrated that the red fluorescence signal from the amNRs was confined within the blood vessels at 60 min. When the cylindrical magnet was applied to the tumor, the GMF gradually drove the extravasation of the amNRs from the blood vessels to deeply penetrate into the surroundings. To further investigate this phenomenon, tumor‐bearing mice were administrated with DiD‐labeled amNRs via intravenous injection, and a magnet was fixed at the tumor site (amNR + GMF group) or not (amNR group). At 2 h postinjection, the blood vessels of treated mice were stained with DyLight‐488‐conjugated tomato lectin. Then, the spatial distribution of amNRs in the tumor tissue was observed by a Z‐series of confocal laser scanning microscope (CLSM) (Figure 3k). Notably, the amNRs (red) were mainly located in the perivascular area (green) in the amNR group, while red fluorescence signals were uniformly distributed throughout the tumor tissue in the amNR + GMF group, indicating efficient deep penetration into tumor tissue under GMF. Moreover, the tumor tissues of the treated mice were harvested, sectioned, and stained with anti‐CD31 antibodies for CLSM observation (Figure 3l). Similar to the previous observations, the red fluorescence attributed to the amNRs had perfused throughout the whole tumor tissue in the amNR + GMF group, even in locations far from blood vessels. In contrast, without GMF application (amNR group), the red fluorescence signals were mainly confined to the area around the blood vessels.

In addition, we evaluated the effect of the specific distance between the magnet and tumor mass on the penetration of amNR (Figure S9a, Supporting Information). As shown in Figure S9b,c (Supporting Information), the area proportion of red fluorescence signal (amNR) decreased with increasing distance between the magnet and tumor. As the distance from the magnet increased, the magnetic force on amNR decreased gradually (Figure 3h). Next, we explored and evaluated the influence of the magnet's orientation on the tumor penetration of amNR (Figure S10a, Supporting Information). As shown in Figure S10b,c (Supporting Information), efficient deep penetration of amNR into tumor tissue was observed when the underside of the magnet faced the tumor site. In contrast, when the flank of the magnet faced the tumor site, the tumor penetration of amNR was significantly impaired. It might be attributed to that the magnetic field intensity near the flank of the magnet is lower than that near the underside (Figure 3g).

### 2.3 The amNRs Possess A Recoverable TAT Ligand for Active Tumor Cell Uptake

**Figure 4:**
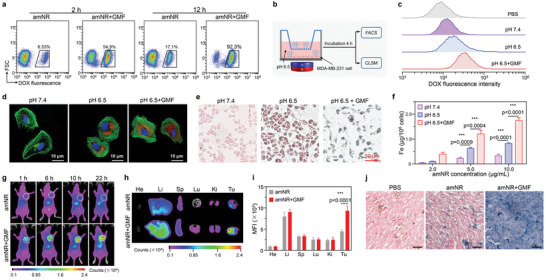
The acidic microenvironment of deep tumor tissue uncovered the masked TAT ligand on the amNRs for active tumor cell uptake. a) Flow cytometry analysis of the percentages of DOX‐positive MDA‐MB‐231/GFP cells at 2 and 12 h postinjection of amNRs. b) Schematic illustration of the cellular uptake of amNRs under a GMF at pH 6.5 in the Transwell system. c,d) Flow cytometry analysis (c) and CLSM images (d) of MDA‐MB‐231 cell uptake of amNRs at pH 6.5 under a GMF. Coincubation of MDA‐MB‐231 cells and amNRs at pH 6.5 and pH 7.4 were used as controls. e) Cellular uptake of amNRs investigations using nuclear fast red and Prussian blue double‐staining. f) Inductively coupled plasma‐mass spectrometry (ICP‐MS) analysis of Fe content in MDA‐MB‐231 cells under the treatments shown in (b) Data are shown as mean ± S.D. n = 3. One‐way ANOVA with a Tukey post hoc test. ***p < 0.001. g) In vivo distribution of DiD‐labeled amNRs after intravenous injection with or without GMF application. h) Representative ex vivo imaging of the major organs (He: heart, Li: liver, Sp: spleen, Lu: lung, and Ki: kidney) and tumor (Tu) tissues after administration of amNRs 24 h. i) Region of interest (ROI) analysis of the fluorescence signals in the collected tissue in (h). Data are presented as the mean ± S.D. n = 3. Student's *t*‐test. ***p < 0.001. j) Distribution and accumulation of amNRs in the tumors determined by Prussian blue staining at 24 h postinjection (scale bar, 50 µm).

The amNRs were decorated with a masked TAT ligand, whose targeting ability would be recovered in the acidic tumor microenvironment for active tumor cell uptake. To demonstrate this hypothesis, we established an MDA‐MB‐231/GFP tumor xenograft model (Figure S11, Supporting Information). Mice with or without magnet placement at the tumor site were administrated with amNRs as described above. In the amNR + GMF group, DOX was detected in 54.9% of tumor cells at 2 h postinjection (**Figure** **4**a), and the percentage of DOX‐positive cells reached 92.3% at 12 h postinjection. In stark contrast, the proportions of DOX‐positive cells in the amNR group were only 6.53% (2 h postinjection) and 17.1% (12 h postinjection). This significant difference in cellular uptake should be attributed to the efficient targeting of the uncovered TAT peptide via the magnetic field‐driven penetration of amNRs into deep tumor tissue. Because the extracellular pH in tumors decreases as the distance to blood vessels increases,^[^ ^27^, ^28^, ^29^ ^]^ the enhanced tumor extracellular acidity would more efficiently uncover the masked TAT ligand (Figure S12, Supporting Information). To confirm the above speculation, we designed an in vitro Transwell system with magnet placement (Figure 4b) for validation. amNRs were added to the upper chambers, and tumor cells were cultured in the lower chambers. Flow cytometric analysis clearly demonstrated that the intracellular DOX signals were the strongest in the amNR + GMF group at pH 6.5 (Figure 4c), which was further corroborated by CLSM observations (Figure 4d). Furthermore, Prussian blue iron staining (Figure 4e) and intracellular iron content analyses (Figure 4f) also showed that the uptake of amNRs by tumor cells was the highest under a GMF at pH 6.5. In addition, the TAT reactivation (amNR + pH 6.5 group) also affected the endocytosis pathway of amNR compared to that before TAT reactivation (Figure S13, Supporting Information).

Benefiting from the active penetration and cellular uptake of the amNRs under a GMF, the final retention of amNRs in tumor tissue should be enhanced. To confirm it, the fluorescence signals of DiD‐labeled amNRs were measured using a Bruker In Vivo Xtreme imaging system. Obviously, the final tumor retention of amNRs was significantly increased under a GMF at all time points (Figure 4g). Ex vivo imaging and fluorescence signal analysis of ROI (Figure 4h,i) demonstrated that the fluorescence signals in the amNR + GMF group were ≈2.1‐fold higher than those in the amNR group. In addition, Prussian blue iron staining of collected tumor slices confirmed that the tumor accumulation of amNRs was significantly improved under a GMF (Figure 4j).

### 2.4 amNRs Rapidly Released the Encapsulated DOX via Application of an AMF

**Figure 5:**
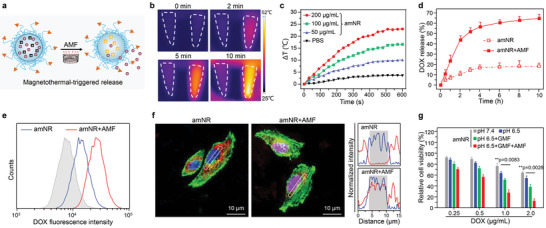
The amNRs exhibit active DOX release after AMF treatment. a) Schematic illustration of active DOX release from the amNRs via AMF‐mediated heat generation. b) Thermographic maps of the centrifuge tubes containing PBS (1 mL, left) or amNRs (1 mL, [Fe] = 100 µg mL^−1^, right) subjected to AMF (25 kA m^−1^, 317 kHz). c) Time‐dependent temperature change curves of amNRs ([Fe] = 200, 100, 50 µg mL^−1^) under an AMF for 10 min. d) DOX release profiles from amNRs with or without AMF treatment. Data are presented as the mean ± S.D. (n = 3). e) Flow cytometric analyses of MDA‐MB‐231 cells after AMF treatment and further incubation for 4 h. The MDA‐MB‐231 cells were precultured with amNR for 4 h. f) CLSM images of intracellular release in the Transwell system (e) for 4 h followed by treatment with an AMF (25 kA m^−1^, 317 kHz) for 10 min. The colocalization of amNRs (red line) with the nucleus (blue line) is shown in the right section. g) Viability of MDA‐MB‐231 cells after incubation with amNRs in the Transwell system (e) under different conditions. Data are presented as the mean ± S.D. (n = 3). One‐way ANOVA with a Tukey post hoc test was performed in (g). **p < 0.01.

The amNR core, which consisted of soft PPE with a very low *T*_g_, not only endowed distinctive deformability as described above but also possessed heat‐induced supersensitive drug release potency (**Figure** **5**a).^[^ ^13^ ^]^ Additionally, the FN inside the amNR core has an excellent heat conversion effect (Figure 5b,c) under an AMF (25 kA m^−1^, 317 kHz), which could achieve AMF‐driven drug release. Moreover, after AMF application for 5 cycles, the process of temperature elevation did not significantly change, suggesting satisfactory heat conversion stability of amNR under AMF (Figure S14, Supporting Information). As expected, Figure 5d demonstrates that ≈60% of the DOX was released from the amNRs after AMF treatment (amNR + AMF group), while only ≈16% of the DOX was released from the amNRs without AMF treatment.

Moreover, to investigate whether AMF‐driven active drug release can be achieved within tumor cells, we incubated MDA‐MB‐231 cells with amNRs under a GMF at pH 6.5 for 4 h followed by exposure to an AMF for 10 min (Figure S15, Supporting Information). Because the fluorescence emission of DOX had the characteristics of aggregation‐caused quenching, the controlled release of amNR would lead to the enhancement of the DOX fluorescence signal.^[^ ^23^ ^]^ As shown in Figure 5e, compared with the group without AMF treatment (amNR), the intracellular DOX fluorescence signal of the amNR + AMF group was significantly enhanced, indicating that amNR achieved AMF‐driven drug release. CLSM was employed to detect the intracellular DOX distribution after further incubation for 4 h (Figure 5f). Strong red fluorescence signals from DOX were found in the cell nuclei after AMF treatment, indicating efficient intracellular DOX release. In contrast, without AMF treatment (amNR group), the DOX fluorescence signal remained localization in the cytoplasm. In addition, the co‐localization results of amNR and lyso/endosome also showed that DOX was released after AMF treatment and then transported into the nucleus (Figure S16, Supporting Information).

As described above, amNRs achieved active penetration, cellular uptake, and intracellular drug release through magnetic stimulation. Such active drug transport throughout the entire delivery process would improve therapeutic efficacy, and this was first evaluated. After treatment as mentioned in Figure S15 (Supporting Information), MDA‐MB‐231 cell viability was detected by employing a standard MTT assay. As shown in Figure 5g, amNR treatment exhibited the lowest cell viability under a GMF (active penetration) at pH 6.5 (active cellular uptake) with subsequent AMF application (active drug release), which fully illustrated the advantage of whole‐process active drug transport in cancer therapy.

### 2.5 Antitumor Efficacy of Magnetically Driven amNRs

**Figure 6:**
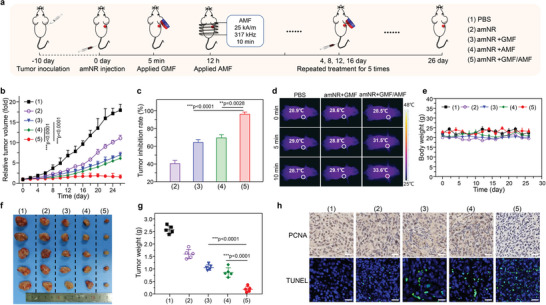
amNRs, with whole‐process active drug transport capability, achieved complete tumor growth inhibition. a) Schematic illustrating the treatment procedure. b) Tumor volume curves generated from the mice treated with PBS, amNRs, amNRs + GMF, amNRs + AMF, and amNRs + GMF/AMF. Data are presented as the mean ± S.D. n = 5. c) Tumor inhibition rates of various groups. d) Thermal images of mice after administration of amNRs upon AMF induction. e) Body weights of the treated mice at different time points (n = 5). f,g) Digital photographs (f) and weights (g) of the collected tumor masses at the end of treatment. Data are presented as the mean ± S.D. (n = 5) in (b,c,g). One‐way ANOVA with a Tukey post hoc test. *p < 0.05, ***p < 0.001. h) PCNA and TUNEL staining of the tumor tissues. Scale bars: 50 µm.

Encouraged by the active drug transport throughout the entire delivery process, the therapeutic efficacy of amNRs was evaluated in the MDA‐MB‐231 tumor model. Tumor‐bearing mice were subjected to different treatments (**Figure** **6**a): 1) PBS; 2) amNRs; 3) amNRs + GMF; 4) amNRs + AMF; and 5) amNRs + GMF/AMF. As anticipated, the growth of the tumors from mice that were intravenously injected with amNRs and then received GMF/AMF treatment was almost completely delayed (Figure 6b), exhibiting an inhibition rate of 96.1% (Figure 6c), which was notably higher than that of other groups due to whole‐process active drug transport. In addition, the tumor inhibition rates in the amNR, amNR + GMF, and amNR + AMF groups relative to the PBS control group were ≈40.4%, 64.2%, and 69.4%, respectively. It should be noted that the heat generated by the AMF was very weak (Figure 6d) and insufficient to induce a hyperthermic anticancer effect. Additionally, these mice did not exhibit obvious weight change during the entire treatment procedure (Figure 6e), and the hematoxylin and eosin staining (H&E) of main organs also did not show obvious organ injury (Figure S17, Supporting Information), verifying the good safety of the amNRs. At 26 days posttreatment, tumor tissues were collected and weighed. The photographs (Figure 6f) and the average tumor weights (Figure 6g) further indicated the best therapeutic efficacy from amNR administration with the assistance of GMF/AMF. Moreover, to further confirm the therapeutic effects, proliferating cell nuclear antigen (PCNA) and terminal deoxynucleotidyl transferase‐mediated dUTP nick end labeling (TUNEL) staining were conducted. These results demonstrated that the tumors from mice treated with amNR + GMF/AMF had experienced substantial apoptosis and decreased proliferation (Figure 6h).

## 3 Discussion

Currently, a dozen nanomedicines have been approved as delivery systems for anticancer agents in the clinic.^[^ ^30^, ^31^ ^]^ However, the anticancer efficacies of these approved nanomedicines are still unsatisfactory and have caused many critics to question their utility.^[^ ^4^, ^5^ ^]^ For instance, Wilhelm et al. concluded that only 0.7% of these nanomedicines were delivered into tumor tissue according to the data of 117 published preclinical studies.^[^ ^32^ ^]^ One key theme in the field of nanomedicine is how to increase drug delivery efficacy. Indeed, low delivery efficacy is essentially due to the passive drug transport performance of these approved nanomedicines, which results in their inability to cross a series of biological barriers throughout the entire delivery process.^[^ ^5^, ^33^, ^34^ ^]^

To address the aforementioned issue, we developed a magnetically driven amoeba‐like nanorobot, amNR, to achieve whole‐process active drug transport. The core of the amNRs, which consisted of PPE with a *T*_g_ of −76.8 °C (Figure 3a), endowed the nanomaterial with its distinctive deformation capability. As a result, the amNRs with a size of ≈185 nm can cross 100, 80, and even 50 nm interstitia (Figure S4, Supporting Information). Furthermore, the amNRs achieved active extravasation and deep penetration into tumor tissue under a GMF (Figure 3j–l). Then, the acidic microenvironment of the deep tumor site uncovered the masked TAT ligand for active tumor cell uptake (Figure 4c–f). Accordingly, the DOX was delivered into ≈92.3% of tumor cells through the cascade of active penetration and cellular uptake (Figure 4a), and DOX accumulation in tumor tissue was also notably increased by 2.1‐fold (Figure 4g–i).

Moreover, the encapsulated FN in the amNRs generated heat under an AMF, which triggered supersensitive DOX release (Figure 5d) within tumor cells (Figure 5e,f). Such drug release is attributed to the very low *T*_g_ of the PPE in the core of the amNRs. Eventually, the amNRs achieve whole‐process active drug transport through magnetic stimulation and their own deformability, completely delaying tumor growth with an inhibition rate of 96.1% (Figure 6b,c).

In addition, there are many studies show that soft nanoparticle consume more energy in the cellular uptake process than a hard nanoparticle.^[^ ^35^, ^36^ ^]^ This makes it uneasier for cells to ingest soft nanoparticles. Therefore, the deformation characteristics of amNR promote its deep penetration in tumor tissue, but there are indeed drawbacks in the uptake of tumor cells. To address this drawback, in this study, the TAT peptide was used to decorate the soft PPE‐based nanoparticles to enhance cellular uptake.

In conclusion, we constructed an amNR that achieved active drug transport to conquer a series of biological barriers. The distinctive deformation capability of the amNRs ensured active extravasation and penetration into deep tumor tissue under a GMF, its masked TAT ligand was uncovered for active tumor cell uptake, and the heat generated by FN under an AMF improved the release of intracellular DOX. As a result, the whole‐process active drug transport of the amNRs completely inhibited tumor growth. Overall, our work offers a strategy to actively transport therapeutic agents throughout the entire delivery process for nanomedicine‐mediated cancer therapy.

## 4 Experimental Section

### Materials

The ^DA^TAT‐PEG‐*b*‐PPE was synthesized according to the literature.^[^ ^23^ ^]^ The maleimide‐terminated diblock polymer Mal‐PEG‐*b*‐PPE was first synthesized by polymerization of monomers 2‐hexoxy‐2‐oxo‐1,3,2‐dioxaphospholane using maleimide‐terminated PEG (Mal‐PEG‐OH, Mn = 3400 Da, Tansh‐Tech Co., Ltd., Guangzhou, China) as a macroinitiator. Then, the TAT peptide TAT (YGRKKRRQRRRC, Mr = 1663) was chemically conjugated to Mal‐PEG‐*b*‐PPE through a thiol‐maleimide reaction; and subsequently, 2,3‐dimethylmaleic anhydride (DA) was used to modify the lysine residues’ amines of TAT peptide to obtain ^DA^TAT‐PEG‐*b*‐PPE. Ferrimagnetic nanocube (FN) was synthesized following the method described in the previous report.^[^ ^37^ ^]^

### Determination of Glass Transition Temperature (T_g_) and Young's Modulus

The glass transition temperatures (*T*_g_s) of polyphosphoester and poly(lactic acid) were measured by DSC on NETZSCH DSC 214 Polyma (Germany). Briefly, 10 mg of the polymer was placed in a crucible and heated at a heating rate of 10 °C min^−1^ under N_2_, and the DSC traces at the second heating was monitored to calculate the *T*_g_.

The Young's modulus of polyphosphoester and poly(lactic acid) was quantified by atomic force microscope on Asylum MFP‐3D SA (Santa Barbara, USA). The test process was performed with silicon nitride tips at a trigger voltage of 0.5 V. Young's modulus was determined by fitting the force‐distance curve by the Hertz equation.^[^ ^38^ ^]^

### The Transportation of amNR with PPE Core Through Membrane Filters

A self‐made Transwell system with a 100 nm pore‐size membrane filter was used to further explore the penetration efficiency of amNR driven by magnetic force. Briefly, 0.1 mL of amNR solution ([DOX] = 100 µg mL^−1^) was placed in the upper chamber of Transwell, and 1 mL of water was added to the lower chamber. After placing the cylindrical permanent magnet under the lower chamber, 0.1 mL of solution in the lower chamber was collected at 2, 4, 8, 12, and 24 h, and then 0.1 mL of deionized water was supplemented. The content and size distribution of amNR passing through the filter membrane (amNR in the lower chamber) was detected by fluorescence spectrophotometer and dynamic light scattering respectively. The control group was treated with the same experimental scheme as above, except for placing a magnet. In addition, Transwell was placed at a different distance from the cylindrical magnet, and the transportation efficiency of amNR to the membrane filter was detected according to the above method.

### Drug Release Triggered by Magnetic Hyperthermia

The amNR solution (1 mL, [Fe] = 100 µg mL^−1^) was treated with an alternating magnetic field (AMF, H = 25 kA m^−1^, *f* = 317 kHz) generated by high‐frequency induction heating equipment for 10 min. In the control group, an equal volume of PBS underwent the same treatment. The change in real‐time temperature of the sample solution was monitored by an infrared camera (ICI7320, Infrared Camera Inc., USA). In addition, the temperature change of the amNR solution (1.0 mL, [Fe] = 100 µg mL^−1^) over five on/off cycles under AMF application was measured to evaluate its heat conversion stability. In vitro release of DOX from amNR ([DOX] = 100 µg mL^−1^, [FN] = 100 µg mL^−1^) was performed in PBS under mild shaking in a 37 °C water bath. After AMF treatment for 10 min, the PBS outside dialysis bag (MWCO 14 000 Da) was replaced by an equal volume of fresh PBS buffer at predetermined intervals, and the DOX content in the collected external PBS was detected by fluorescence spectrophotometer using a standard curve method.

### In Vitro Tumor Cell Uptake Analysis

MDA‐MB‐231 cells were inoculated in the lower chamber of Transwell (pore size, 0.4 µm) at a density of 5 × 10^4^ per well. Then amNR (0.1 mL, [DOX] = 50 µg mL^−1^) was added to the upper chamber. Meanwhile, the medium of the lower chamber was replaced with 0.5 mL of DMEM medium (pH 7.4 or 6.5). A cylindrical permanent magnet was placed below the Transwell plate for the magnetically actuated treatment. After 4 h of incubation, these samples were harvested for flow cytometry analysis. In addition, cell internalization of amNR was further visualized by CLSM. Moreover, Nuclear fast red and Prussian blue double staining of MDA‐MB‐231 cells was also performed to evaluate tumor cell uptake. To study the uptake mechanism, MDA‐MB‐231 cells were pre‐incubated with various inhibitors including Methyl‐*β*‐cyclodextrin (5.0 µm), chlorpromazine (10 µg mL^−1^), and amiloride (50 µm). The incubation was at 37 °C and for 1 h prior to treatment with amNR or amNR + pH 6.5 for another 4 h. Subsequently, the cells were washed twice and trypsinized for flow cytometry analyses.

### Intracellular DOX Release Under AMF

To determine the AMF‐responsiveness of amNR, the intracellular DOX release at different conditions was also performed. A solution of amNR (0.1 mL, [DOX] = 50 µg mL^−1^) was added to the upper chamber of Transwell which was inoculated with MDA‐MB‐231 cells. After magnetically actuated treatment for 4 h, Transwell‐Plate was put inside the copper coil with AMF (H = 25 kA m^−1^ and *f* = 317 kHz) for 10 min. After further incubation for 4 h, the cells were harvested for flow cytometry and CLSM analyses. For co‐localization analysis of amNR and lyso/endosome, the nucleus and the lyso/endosome were stained by 10 µg mL^−1^ DAPI and 100 nm LysoTracker Green DND‐26 (Thermo Fisher Scientific, Germany). And, the samples were used for CLSM observation. In addition, a standard MTT assay was performed to determine the cell viability after further incubation for 20 h.

### Pharmacokinetics Study

Female ICR mice were administrated with free DOX or amNR at an equivalent DOX injection dose of 5 mg kg^−1^. At predetermined time points, 100 µL of blood was collected from the treated mice, and then heparinized and centrifuged to collect the plasma. Then the DOX was extracted with chloroform/acetonitrile component solvent (1.0 mL, 4:1, v/v), and the organic phase was dried under the nitrogen stream. Finally, the obtained residues were redissolved in 200 µL of DMSO and the DOX content in the plasma was detected by ultra‐performance liquid chromatography.

### Tumor Penetration Study

For the real‐time intravital observation, a dorsal skin fold window chamber model of MDA‐MB‐231tumor bearing mice was constructed. Briefly, the mice were anesthetized with an injection of 1% pentobarbital sodium into the abdominal cavity. Subsequently, the small dorsal kit (SM100, APJ TRADING CO., INC.) was fixed on the mouse dorsal skin with screws and suture. Then, a circular incision was made inside the fixture and the skin flap was cut off without injuring the vessels. After injecting 5 × 10^5^ MDA‐MB‐231 cells into the submucosa, the cover slip (12 mm) was used to cover the incision. The mouse was placed on a thermoplate until they woke up. On the seventh day after model construction, amNR was injected 1 h before anesthesia via a caudal vein. Subsequently, the mice were placed on an electric stage of a spinning‐disk confocal microscope for detection. All intravital observations were performed using an OLYMPUS SpinSR10 microscope.

For the immunofluorescence assay, tumor‐bearing BALB/c nude mice were treated with amNR, amNR + GMF. The fluorescent‐labeled amNR was prepared by replacing DOX with DiD for tumor penetration study. The administered dose of DiD was equivalent to these formulations (0.5 mg kg^−1^). For the magnetically actuated treatment (amNR + GMF), the magnet was pasted on the tumor site of nude mice with breathable pressure‐sensitive adhesive tape (Qingdao Hainuo Biological Engineering Co., Ltd.) after intravenous injection. Two hours later, the mice were sacrificed to excise tumor tissue, then rinsed with PBS and subjected to cryotomy. The tumor tissues were sectioned into slides of 8‐µm thickness. The frozen tumor sections were labeled with Anti‐CD31 Rabbit pAb primary antibody and then Alexa Fluor 488‐conjugated Goat Anti‐Rabbit IgG secondary antibody. Then the tumor sections were observed under the Nikon Ti‐E A1 CLSM system with a 20× objective. For exploring the influence of the distance between the magnet and the tumor, only the distances were adjusted at the stage of applying GMF, and the other processes were the same as above.

For the 3D image of tumor sections, after underwent similar treatment as above, tomato lectin conjugated with DyLight 488 was intravenously administrated, the mice were sacrificed at 5 min postinjection, and the tumors were excised, fixed in 4% paraformaldehyde, and dehydrated in 30% sucrose solution. The tumors were then immersed in O.C.T. solution and subjected to cryotomy to collect 100 µm thickness serial sections. The samples were observed by the Z‐series of CLSM.

### Tumor Cell Uptake Assay In Vivo

MDA‐MB‐231/GFP tumor model was first established. After setting up the above‐mention experimental group (amNR, amNR + GMF), tumor tissues were excised at 2 and 12 h postinjection and digested with 0.1% collagenase I into single cells. MDA‐MB‐231/GFP was isolated by flow cytometry and the proportion of DOX‐positive cells was detected.

### Biodistribution Assays

The tumor‐bearing nude mice were treated with amNR and amNR + GMF as described above. At predetermined time intervals, the in vivo fluorescence distribution in the mice was detected by Bruker In Vivo Xtreme. At 24 h postinjection, the mice were sacrificed, and then the tumor tissues and main organs were collected and imaged by Bruker In Vivo Xtreme. In addition, at 24 h post‐injection, the tumor tissues of each group were isolated and counterstained with Nuclear fast red and Prussian blue. Then, the stained tumor tissue sections were evaluated by a digital pathological scanning system (Aperio CS2, Leica).

### In Vivo Tumor Growth Inhibition

For the tumor suppression study, the tumor‐bearing BALB/c nude mice (n = 5) were randomly treated with PBS, amNR, amNR + GMF, amNR + AMF, or amNR + GMF + AMF at the equivalent DOX dose of 5.0 mg kg^−1^. For the magnetically actuated treatment, a magnet was pasted on the tumor site after intravenous injection for 12 h. For the treatment of AMF, the mice were put inside the copper coil and treated with AMF (H = 25 kA m^−1^, *f* = 317 kHz) excited by field coils for 10 min. Mice received these treatments once every four days. Tumor volume (mm^3^) was calculated as V = ab^2^/2, in which a and b indicate the length and width of the tumor. After 26 days of treatment, All the mice were sacrificed and tumor tissues were excised to measure the weight.

### Statistical Analysis

All data were shown as means ± standard deviations (s.d.). To measure significant differences among the treatment groups, a one‐way analysis of variance (ANOVA) followed by the Tukey post hoc test was used for multiple comparisons, and Student's *t*‐test was used for two‐group comparisons. . *p < 0.05 was considered to be statistically significant, **p < 0.01 and ***p < 0.001 were considered to be highly significant. Data were evaluated and compared using GraphPad Prism 7 (GraphPad Software, Inc.).

## Conflict of Interest

The authors declare no conflict of interest.

## Supporting information

Supporting Information

Supplemental Movie 1

Supplemental Movie 2

## Data Availability

The data that support the findings of this study are available from the corresponding author upon reasonable request.
