# Supplementary material for: A Magnetically Driven Amoeba‐Like Nanorobot for Whole‐Process Active Drug Transport
Source: Adv Sci (Weinh). 2023 Jan 25;10(7):2204793. doi: 10.1002/advs.202204793 (PMC9982561; doi:10.1002/advs.202204793)
Supplement: Supplementary file 1 — Supporting Information [file ADVS-10-2204793-s002.pdf]

Supporting information

A magnetically driven amoeba-like nanorobot for whole-process active drug transport

Yueqiang Zhu[1,2,5], Yonghong Song[3,5], Ziyang Cao[1,4]✉, Liang Dong[3], Song Shen[1], Yang Lu[3]✉, Xianzhu Yang[1,2,3]✉

Reagents. Doxorubicin hydrochloride (DOX·HCl) was bought from Dalian Meilun Biotechnology Co., Ltd. (Dalian, China) and treated with triethylamine to obtain hydrophobic DOX. DiD, DAPI, MTT, and Alexa Fluor® 488 phalloidin were purchased from Thermo Fisher Scientific Inc. (USA). Anti-CD31 rabbit pAb primary antibody and Alexa Fluor® 488-conjugated goat anti-rabbit IgG secondary antibody were purchased from Wuhan Servicebio Technology Co., Ltd. Nuclepore polycarbonate track-etch membrane filters (Pore size: 100, 80, 50 nm) were purchased from Whatman (UK). Other chemicals and reagents were all of analytical grade and used as received.

Characterization. The size distribution of amNR in aqueous solution were measured by dynamic light scattering (DLS; Malvern Zetasizer Nano S90, England). The morphology of the NPs was investigated using TEM (HT-7700, Hitachi) at an acceleration voltage of 100 kV, and the samples were stained by 2 wt% phosphotungstic acid aqueous solution. The fluorescence spectra and drug loading content (DLC) of DOX was measured on spectrofluorophotometer (λ_{ex} = 460 nm, Shimadzu, Tokyo, Japan) using 1 cm path length quartz cell with the scanning speed

of 2000 nm/s. The concentration of FNs (Fe) was determined using inductively coupled plasma mass spectrometer (ICP-MS; Thermo Fisher Scientific ICap RQ). The UV-vis absorption spectrum of the sample solution was measured on UV spectrophotometer (Shimadzu UV-2600, Japan) by measuring absorbance between 300 nm and 800 nm. Confocal laser scanning microscope (CLSM) was performed on Nikon Ti-E A1 CLSM system. Flow cytometry was performed on BD Accuri™ C6 Plus flow cytometer (BD Biosciences, USA). Cell viability was measured using a multi-plate reader (Cytation 5, Biotek, USA). In vivo fluorescence images were determined by Bruker in-vivo Xtreme II (Bruker, Germany).

Preparation of amNR. To prepare amNR, ^{DA}TAT-PEG-*b*-PPE (10.0 mg), FN (1.0 mg) and hydrophobic DOX (1.0 mg) were dissolved into 2.0 mL dimethyl sulfoxide (DMSO), and then the mixed solution was slowly added dropwise to 10 mL ultrapure water with ultrasonic treatment for 30 min. Subsequently, DMSO in the samples was removed by dialysis overnight in ultrapure water, and the unencapsulated DOX and FN were removed by filtration using a filter membrane (0.45 μm). Finally, the samples were adsorbed and enriched by permanent magnet, and the enriched samples were resuspended in ultrapure water to obtain amNR. In addition, the fluorescent-labeled amNR were fabricated by similar process except for replacing DOX with DiD during the preparation.

Reactivation rate of TAT peptide at tumor acidity. The activation degree of TAT peptide was analyzed by measuring the primary amine concentrations using fluorescamine as the sensor. The prepared amNR (1 mg/mL) were preincubated in

PBS at pH 7.4 or 6.5 for 1 hour. Then fluorescamine solution in DMF (2 mg/mL, 0.2 mL, Shanghai macklin biochemical Co. Ltd., China) was added to samples (1.0 mL) and its fluorescence intensity (Fs, Ex = 365 nm, Em = 475 nm) were measured after 10 minutes incubation. F0 (100% of exposed amine) was defined as the fluorescence intensity of amNR with same concentration after treated with HCl (0.1 M) for 24 h, and Fc was the background fluorescence of PBS. The activation degree of TAT peptide was calculated as following: (Fs−Fc)/(Fo−Fc) × 100%. In addition, the zeta potential changes of amNR were also monitored after incubation at pH 6.5 for 1 h (n = 3).

The transportation of amNR with PPE core through membrane filters. A syringe filtration test, in which amNR were pushed to pass through membrane filters (the filter pores: 100 nm, 80 nm, 50 nm), was adopted for the characterization of amNR mechanical properties and deformation behavior analysis. First, 1 mL of amNR sample ([FN] = 60 μg/mL) was loaded into a gas tight injection needle and extruded through a membrane filter under extrusion force. The real-time change of extrusion force during the extrusion process was monitored by electronic universal testing machine (Instron 5965) at a constant compression speed. Under the maximum extrusion force of 100 N, the filtrate was collected and the iron content was detected by ICP-MS.

Cell lines and animals. The human breast cancer cell line MDA-MB-231 was purchased from the American Type Culture Collection (ATCC, Manassas, VA, USA). The MDA-MB-231/GFP cells were a gift from Professor Jun Wang's Laboratory in

South China University of Technology. And all cells were cultured in Dulbecco's Modified Eagle Medium (DMEM, Gibco, Waltham, MA, USA) containing 10% fetal bovine serum (FBS, Excell Bio, Shanghai, China) and 100 IU/mL penicillin/streptomycin in a humidified atmosphere of 5 % CO_2 at 37 °C.

BALB/c nude female mice and ICR female mice were purchased from the Beijing Vital River Laboratory Animal Technology (Beijing China). All mice were maintained in a specific pathogen-free facility and received human care. All animal experiments were carried out in accordance with the guidelines outlined in the Guide for the Care and Use of Laboratory Animals, and the protocol was approved by the South China University of Technology Animal Care and Use Committee with the assigned accreditation number 2017007.

Tumor inoculation. To establish tumor model, the mammary fat pads of female BALB/c nude mice (6-8 weeks old) was inoculated by 1×10^6 MDA-MB-231 or MDA-MB-231/GFP cells containing 10% Matrigel (BD Biosciences, USA). When the tumor size of the mice reached about 100 mm^3, the tumor-bearing mice were used for subsequent experiments.

Immunohistochemical Analysis. After the last measurement of tumor volume, the mice were sacrificed. Tumor tissues and main organs (heart, liver, spleen, lung, kidney) were immediately excised and fixed in 4% paraformaldehyde overnight and embedded in paraffin. The paraffin-embedded tissues were then cut into about 5.0 μm slices for immunohistochemical analyses. Cell proliferation and apoptosis in tumor tissue were analyzed by immunohistochemical staining of the terminal transferase

dUTP nick-end labeling (TUNEL) assay. The main organs were stained with hematoxylin and eosin (H&E) after dehydration, and the images were recorded on a microscope.

Supplementary Figures

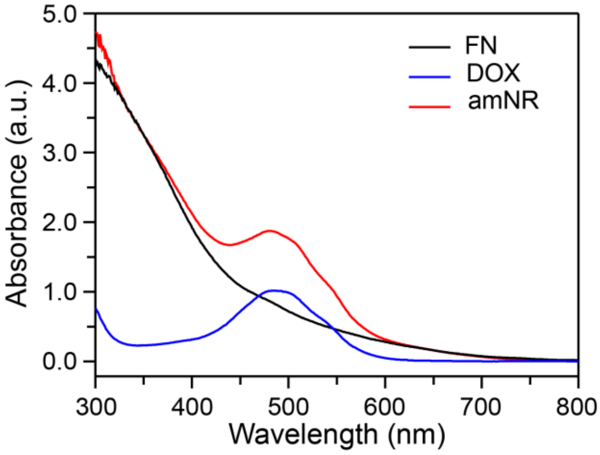

Figure S1. UV-vis absorption spectrum of amNR.

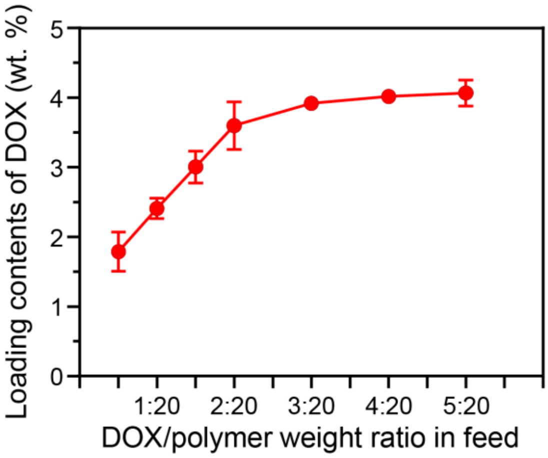

Figure S2. The DOX loading contents for amNR as a function of DOX/polymer feed ratio (wt./wt.).

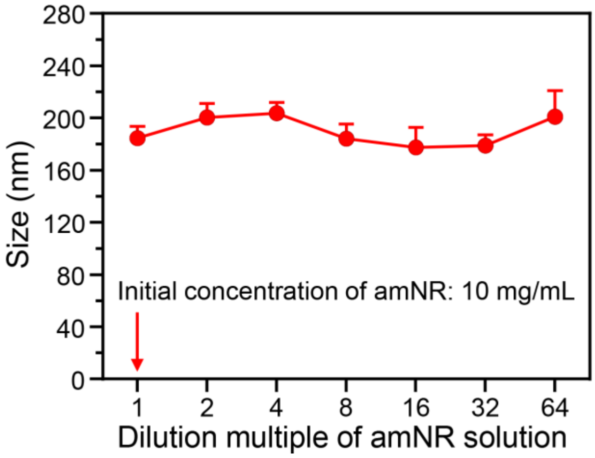

Figure S3. The size changes of amNR at various dilution multiple.

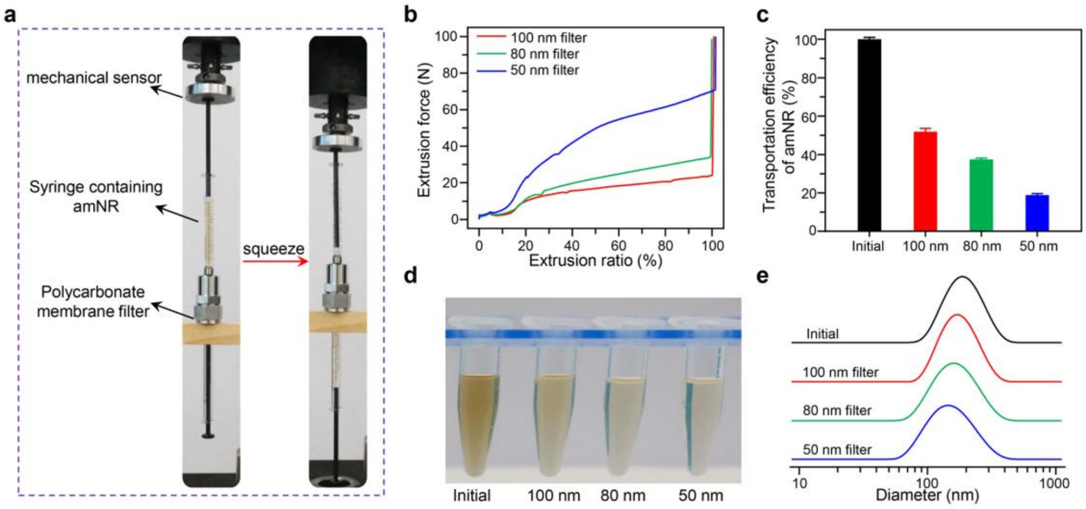

Figure S4. a) Device diagram of the membrane extruder test for amNR (PPE core). amNR were placed in syringe and pushed to pass through membrane filter with different pore size (100, 80, 50 nm). During the extrusion process, the extrusion force change (maximum value 100N) was monitored in real-time by the mechanical sensor, and the iron (Fe) content in the extruded liquid was detected by inductively coupled plasma-mass spectrometry (ICP-MS). b) Real-time monitoring of extrusion force in a membrane filtration process. c) The transportation efficiency of amNR under extrusion force. d) The digital pictures of amNR solution before and after filtration. **e**

The size change of amNR after passing through 100, 80, 50 nm pore size membrane filter.

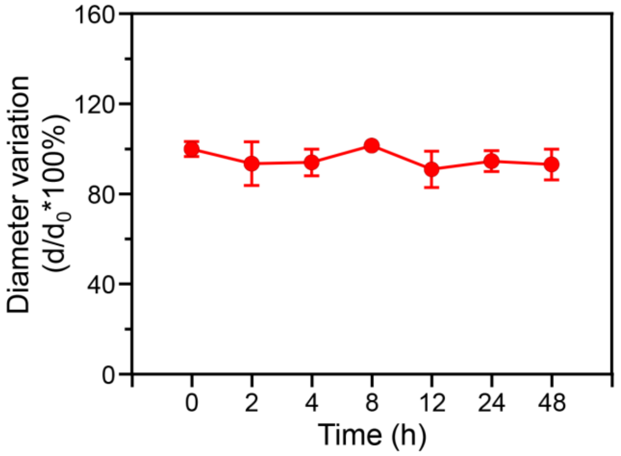

Figure S5. The size stability within 48 h of amNR after passing through 100 nm pore size membrane filter.

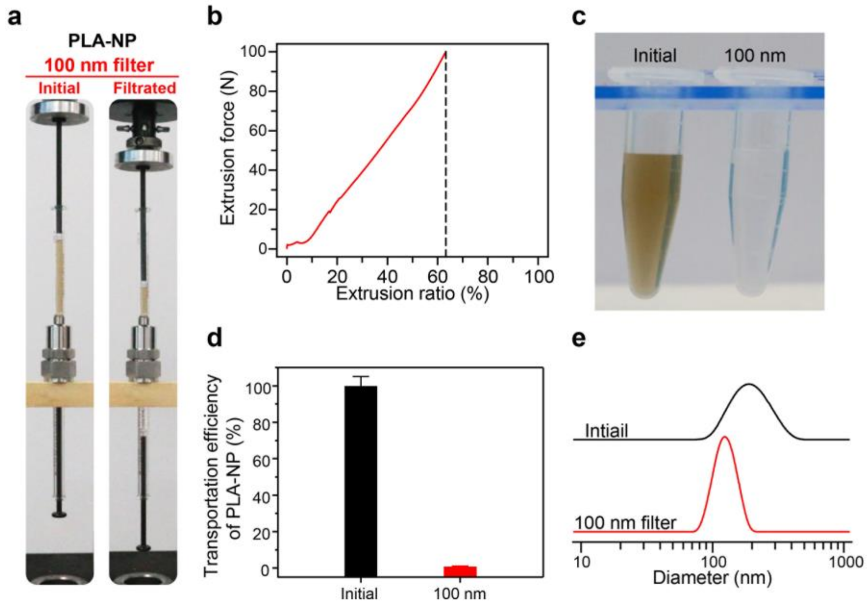

Figure S6. a) Device diagram of the membrane extruder test for PLA-NP (PLA core). b) Real-time monitoring of extrusion force in a membrane filtration process. c) The digital pictures of PLA-NP solution before and after filtration. d) The transportation

efficiency of PLA-NP under extrusion force. e) The size change of PLA-NP after passing through 100 nm pore size membrane filter.

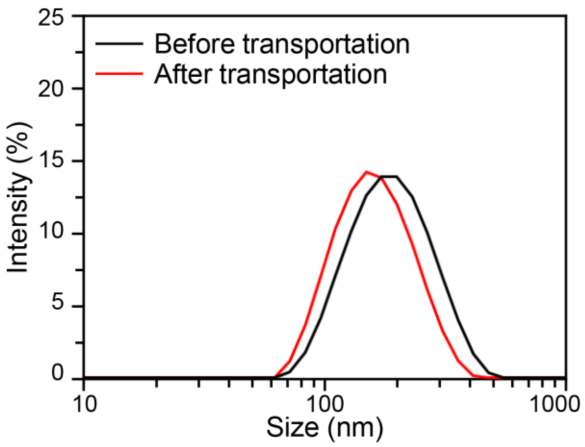

Figure S7. The size change of amNR before and after passing through the 100 nm filter membrane.

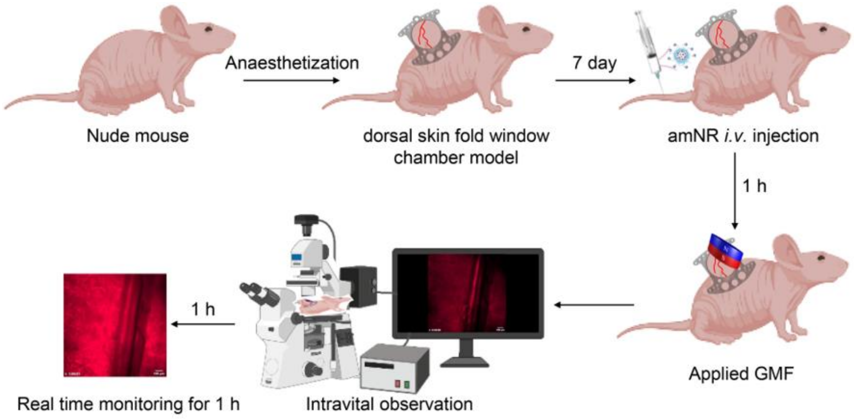

Figure S8. Schematic of the experimental setup and schedule of dorsal skin fold window chamber model for intravital observation.

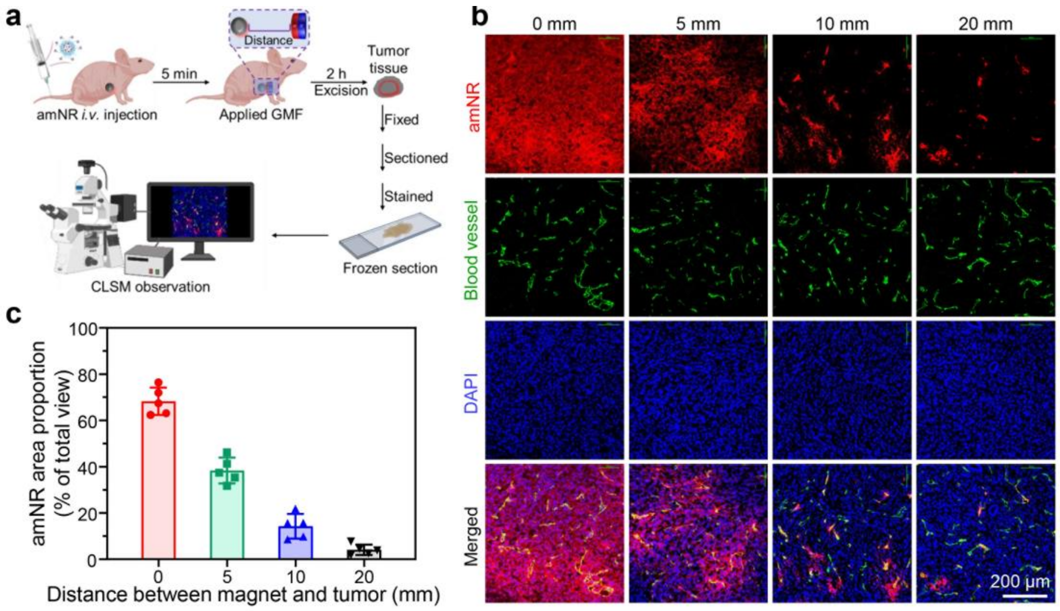

Figure S9. a) Schematic showing the experimental procedure for the tumor penetration of amNR driven by magnets at different distances. b) Observation of the intratumoral distribution of amNR driven by magnets at different distances using a CLSM. Blood vessels: green; Cellular nuclei: blue. c) The area proportion of red fluorescence signal (amNR) under different distance between magnet and tumor. The area proportion of each group was analyzed by Image Pro Plus 6.0 software (n = 5).

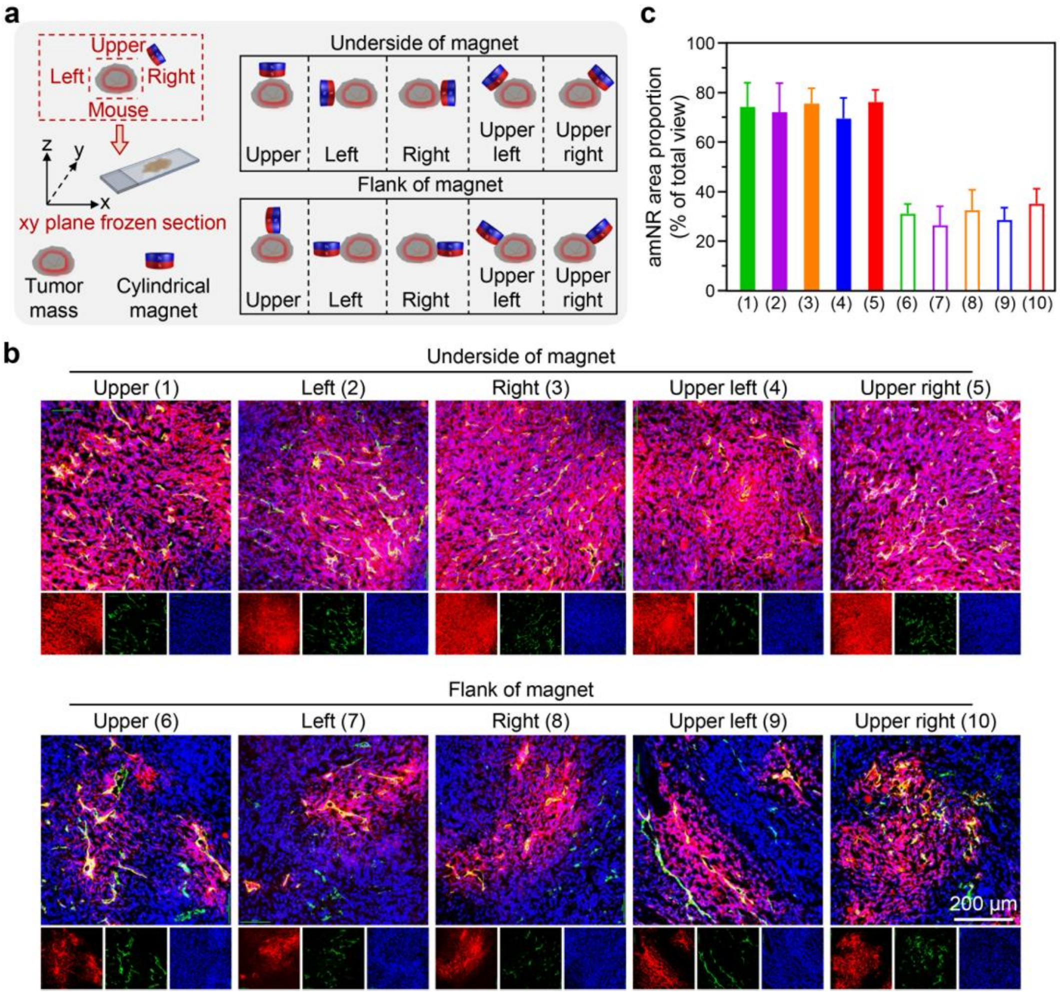

Fig. S10. a) Schematic showing the experimental protocol for the tumor penetration of amNR driven by magnets at different orientation. b) Observation of the intratumoral distribution of amNR driven by magnets at different orientation using a CLSM. Blood vessels: green; Cellular nuclei: blue. The underside or flank of the magnet face to the tumor mass, and the position of magnet relative to the tumor mass was adjusted. After the magnet was placed at different positions and orientations of the tumor site for 2 h, the tumor mass was excised, fixed, dehydrated and sectioned in xy plane at the center. c) The area proportion of red fluorescence signal (amNR) under different orientation between magnet and tumor mass. The area proportion of each group was analyzed by Image Pro Plus 6.0 software (n = 3).

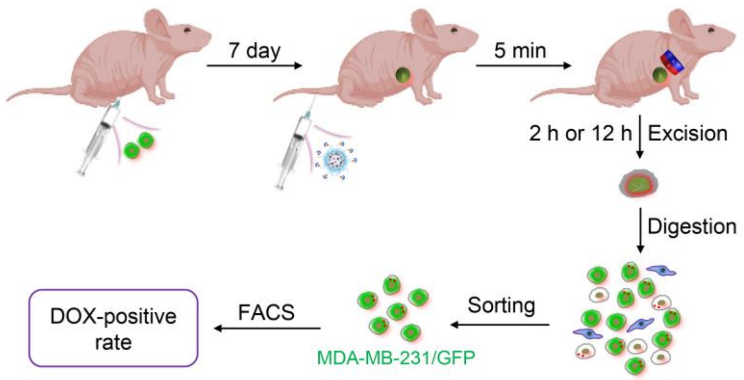

Figure S11. Schematic showing the procedures for analysis of DOX positive rate in the MDA-MB-231/GFP cell.

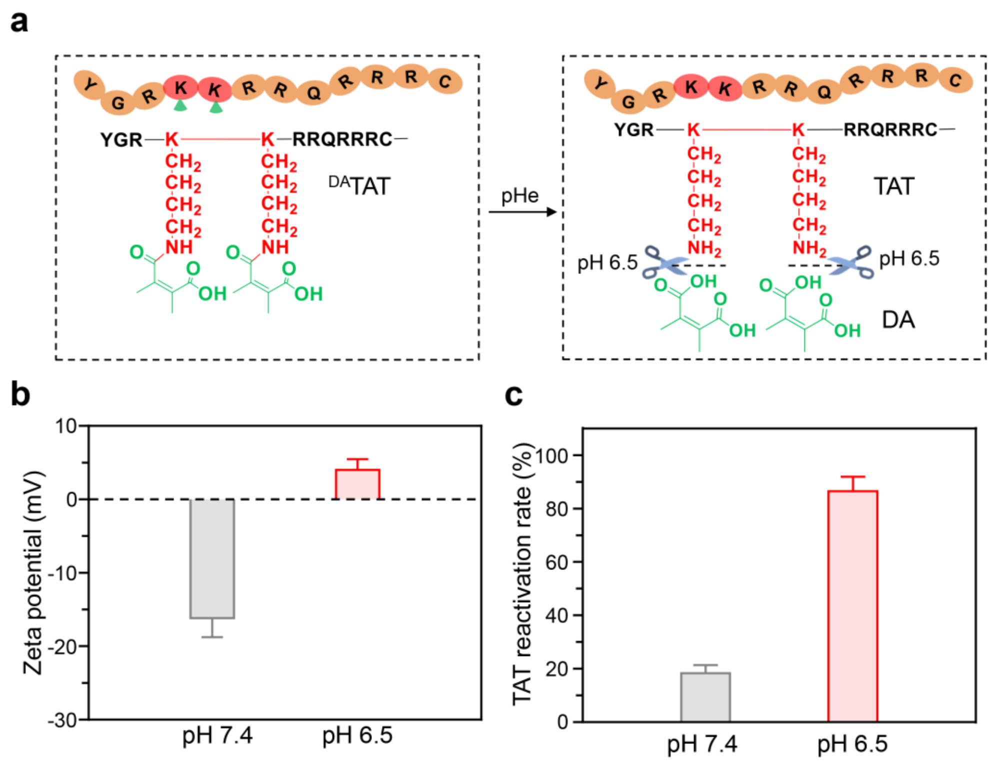

Figure S12. a) Schematic diagram of the reactivation of shielded TAT peptide on the amNR surface under pH 6.5 condition. b) Zeta potential change of amNR at pH 7.4 and 6.5 (n = 3). c) TAT regeneration of amNR at different pH values (n =3). Fluorescamine was used as the sensor.

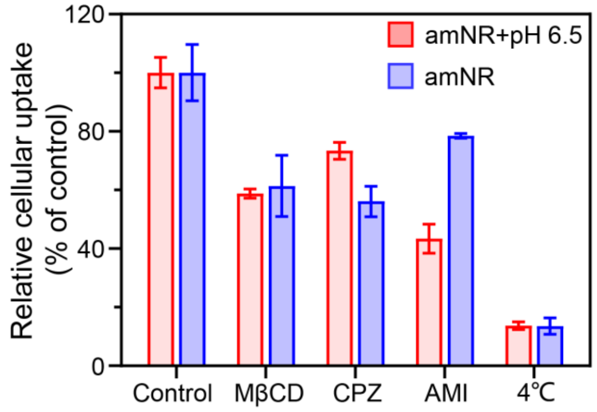

Figure S13. The relative cellular uptake of amNR and amNR+pH 6.5 group in MDA-MB-231 cells after treatment with different inhibitors (MβCD: Methyl-β-cyclodextrin, CPZ: Chlorpromazine, AMI: amiloride).

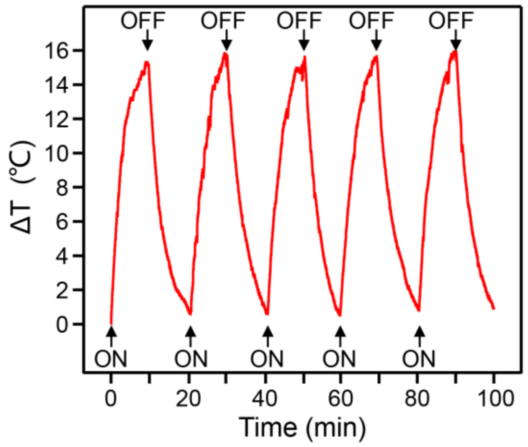

Figure S14. Temperature changes of amNR solution (1.0 mL, [Fe] = 100 μg/mL) over five on/off cycles under AMF application (25 kA/m, 317 kHz).

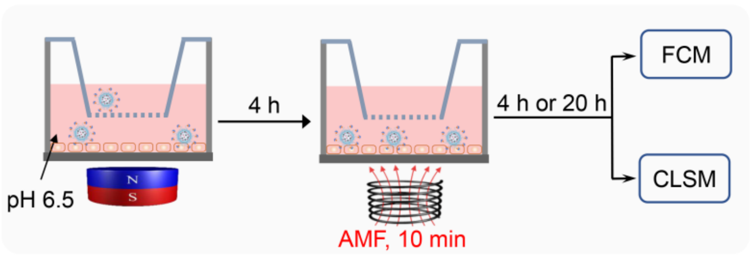

Figure S15. Schematic diagram of the treatment process using the Transwell system to evaluate intracellular DOX release from the amNRs under an AMF.

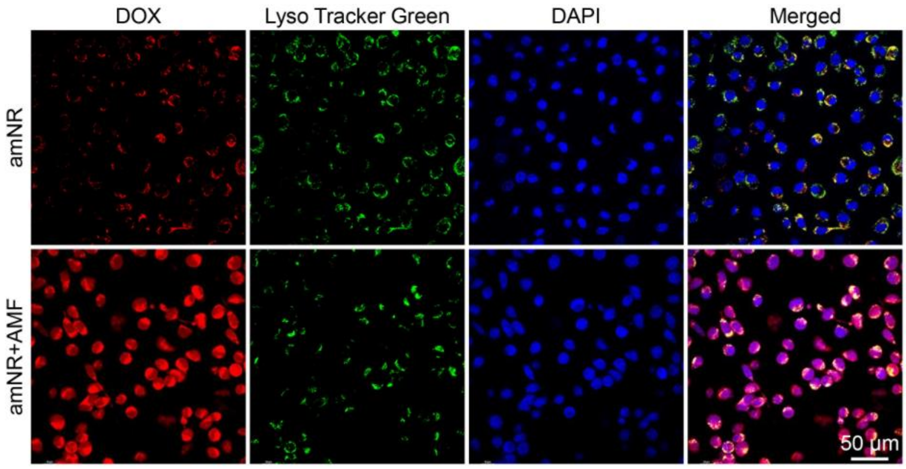

Figure S16. Confocal microscopic images of the intracellular distribution of amNR. The nucleus and the lyso/endosome were stained by DAPI (blue) and LysoTracker Green (green).

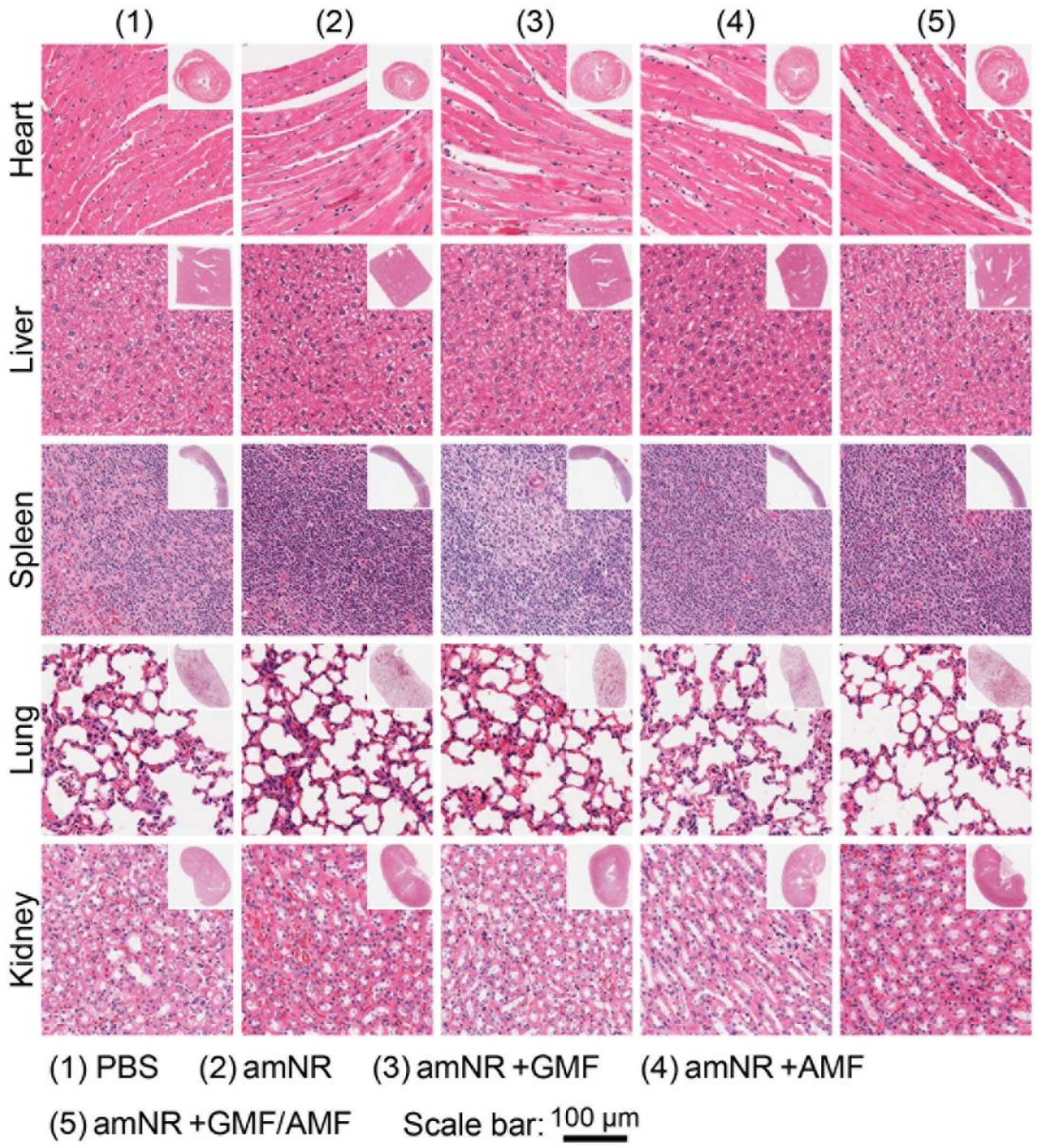

Figure S17. The H&E analysis of main organs (heart, liver, spleen, lung, kidney) after treatment with different formulation.
